# Determination of Fatty Acid Content of Rice during Storage Based on Feature Fusion of Olfactory Visualization Sensor Data and Near-Infrared Spectra

**DOI:** 10.3390/s21093266

**Published:** 2021-05-09

**Authors:** Hongping Lu, Hui Jiang, Quansheng Chen

**Affiliations:** 1 School of Electrical and Information Engineering, Jiangsu University, Zhenjiang 212013, China; lhkingsc@163.com; 2 School of Food and Biological Engineering, Jiangsu University, Zhenjiang 212013, China

**Keywords:** rice, fatty acid, olfactory visualization sensor, near-infrared spectroscopy, feature fusion

## Abstract

This study innovatively proposes a feature fusion technique to determine fatty acid content during rice storage. Firstly, a self-developed olfactory visualization sensor was used to capture the odor information of rice samples at different storage periods and a portable spectroscopy system was employed to collect the near-infrared (NIR) spectra during rice storage. Then, principal component analysis (PCA) was performed on the pre-processed olfactory visualization sensor data and the NIR spectra, and the number of the best principal components (PCs) based on the single technique model was optimized during the backpropagation neural network (BPNN) modeling. Finally, the optimal PCs were fused at the feature level, and a BPNN detection model based on the fusion feature was established to achieve rapid measurement of fatty acid content during rice storage. The experimental results showed that the best BPNN model based on the fusion feature had a good predictive performance where the correlation coefficient (R_P_) was 0.9265, and the root mean square error (RMSEP) was 1.1005 mg/100 g. The overall results demonstrate that the detection accuracy and generalization performance of the feature fusion model are an improvement on the single-technique data model; and the results of this study can provide a new technical method for high-precision monitoring of grain storage quality.

## 1. Introduction

Rice is one of the most important grains in the world, especially in Asia, where rice is the staple food for more than half of the population. Rice is rich in various nutrients needed by the human body; therefore, the quality of rice directly affects human health. China is the world’s largest food producer as well as the largest food consumer [1]. However, due to the seasonality of harvest and the continuity of consumption, in order to guarantee the continuous supply of rice, a certain strategic reserve is still required every year. At present, grain storage is still mainly based on raw grain. In order to respond to emergencies, refined grain storage has become a new development direction for grain storage [2]. Compared with the storage of raw grain, the storage area of refined grain products is smaller and the storage capacity is larger [3]. In case of large-scale natural disasters or public health events, the refined grain products can be quickly mobilized and used, which makes them more flexible. In the fight against coronavirus disease 2019 (COVID-19), the storage of refined grain has played an important role in maintaining food supply.

From processing to consumption of rice, storage is an indispensable intermediate link. During the storage process, the quality of the rice will be reduced. Accordingly, the intrinsic properties of the rice will change more or less during storage [4]. This kind of change is usually called aging. Aged rice is far inferior to fresh rice in terms of color, cooking characteristics and nutritional value. It is a spontaneous and irreversible phenomenon and many factors can cause rice to age [5]. During the storage process, the fat inside the rice is easily oxidized and decomposed to produce free fatty acids, which not only affects the smell of the rice, but also promotes oxidation of the protein and affects the swelling of the starch, which greatly reduces the quality of the rice [6]. Therefore, fatty acid content can be used as an important indicator to measure the storage quality of rice. In general, the fatty acid content of rice will gradually increase with the storage time. However, if the fatty acid content is higher, the quality of the rice will be worse, and if the fatty acid value increases faster, the rice will become moldy [7]. Therefore, the national standard “Guidelines for evaluation of paddy storage character” (GB/T 20569-2006) also uses fatty acid content as the reference basis for judging the storage quality and mildew degree of rice [8]. Although the results from the national standard methods are accurate and reliable, detection personnel must meet high performance requirements and the methods require preparation of special chemical reagents, making the process cumbersome and it is not viable for rapid on-site detection. Therefore, realizing rapid detection of fatty acid content during rice storage is an important practical issue.

Lipids, starch and protein are the most important chemical components in rice. During the storage of rice, lipids change most significantly, followed by starches, with protein changing the slowest. The basic composition of lipids are fatty acids, small molecular compounds composed of C, H, and O. The near-infrared (NIR) spectra are formed by the superposition of the frequency multiplication, combined frequency and difference frequency in absorption bands of the hydrogen group (X-H) in organic substances in the mid-infrared spectrum [9,10,11]. There are a large number of hydrogen-containing groups in fatty acids. Therefore, it is feasible to use the NIR spectroscopy technology to indirectly achieve the rapid determination of the fatty acid value during rice storage. At present, this technology has been widely used for detection and analysis in many fields [12,13,14,15,16,17,18,19], including the detection of food storage quality [20,21,22]. The olfactory sensor is a detection instrument developed to simulate animals’ olfactory organs and can recognize simple or complex odor information volatized by organic substances. Currently, this technology has been successfully applied to detection and analysis in many fields, such as food and agricultural products [23,24,25,26,27,28,29], including successful applications in the detection of food storage quality [30,31,32]. During the storage of rice, due to the loss of the protection of the outer shell, the rice is directly exposed to air and is easily affected by the external environment, resulting in poor storage stability. Therefore, identifying the degree of quality deterioration relates to the safety and utilization of rice storage and has significant economic and social value.

The main cause of rice deterioration is microbial infestation and lipid oxidation. Fatty acid content can be detected to understand the degree of oxidation, thereby further evaluating the freshness of rice. During the storage of rice, the changes in its internal composition are very complicated. However, in the current research on the detection of fatty acid content of rice during storage, most of experiments use single sensor technology. The single sensor detection method often cannot fully reflect the change information in the process, which will affect the reliability of the detection results. In view of this, this study fuses the olfactory sensor and NIR spectroscopy techniques that have been successfully applied in the detection of grain storage quality to establish a more fault-tolerant fusion detection model to achieve rapid detection of rice fatty acid content during storage with high precision.

**Figure 1:**
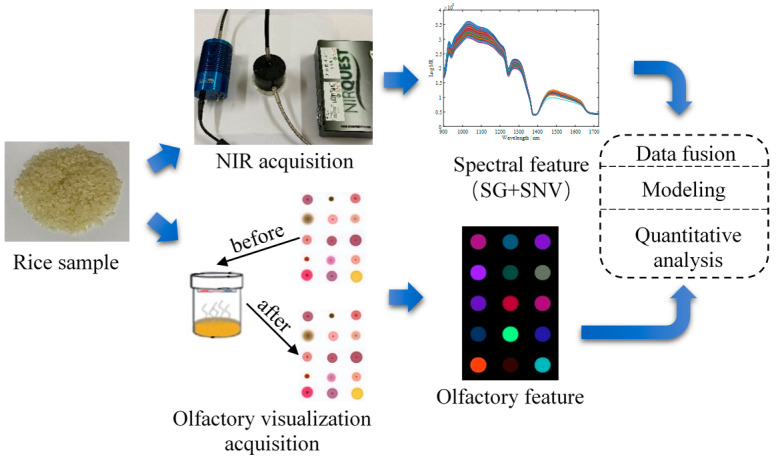
Schematic diagram of detecting fatty acid content of rice during storage based on the fusion of olfactory visualization sensor data and NIR spectra.

Figure 1 shows the main steps used to determine the fatty acid content during rice storage based on the fusion of olfactory visualization sensor data and portable NIR spectra. The specific procedures used are as follows: (1) Preparing a olfactory visualization sensor array as an olfactory sensor system to capture the odor information of rice samples during storage; (2) setting up a portable NIR spectroscopy system to collect NIR spectra of rice samples during storage; (3) pre-processing the obtained olfactory visualization sensor data and portable NIR spectra, and performing principal component analysis (PCA) to extract latent features; (4) optimizing the number of the latent features obtained based on single technical data, and effectively fusing at the feature layer; and (5) establishing a backpropagation neural network (BPNN) model based on the fusion feature to determine the fatty acid content in rice during storage.

## 2. Materials and Methods

### 2.1. Sample Preparation

Rice for the storage experiment was Fulinmen Northeast Rice (China Food Products Marketing Co., Ltd., Shanghai, China) purchased from a local large supermarket (a total of 5 bags and a specification of 5 kg/bag). The purchased rice was stored at 25 °C. Four 20-g (accurate to 0.01 g) rice samples were randomly selected from different locations in each bag, and 20 rice samples were obtained every month. In this way, 160 rice samples were finally obtained in eight months.

### 2.2. Fatty Acid Content Detection

In this study, the national standard (GB/T 20569-2006) was applied to determine the fatty acid value of the rice sample [8]. The main steps are as follows: First, crush the rice and extract the fatty acid in the rice with absolute ethanol; and then add the phenolphthalein reagent, titrate with 0.01 mol/L KOH standard solution, and calculate the fatty acid content according to the amount of KOH solution consumed by the titration. In this study, the fatty acid value of three parallel samples was measured, and the average of the three measurement results was taken as the reference measurement value of the fatty acid value of the sample.

### 2.3. Data Acquisition

#### 2.3.1. Sensor Preparation and Data Collection

The specific preparation process of the sensor was as follows: First, according to the results of the preliminary experiments of our team, the sensor was prepared using 15 color-sensitive materials, including 14 types of porphyrins (Sigma-Aldrich Corp., St Louis, MO, USA) and a hydrophobic pH indicator (Sinopharm Group Chemical Reagent Co., Ltd., Shanghai, China). Then, 8 mg of each color-sensitive material was accurately weighed and added into a 5 mL volumetric flask; the porphyrin material was dissolved in 4 mL of methylene chloride and the pH indicator was dissolved in 4 mL of absolute ethanol. After 30 min sonication, 15 bottles of 2 mg/mL solution were obtained. Finally, the C2 reverse silica gel plate (Merck & Co., Inc., Kenneworth, NJ, USA) was used as the base of the sensor array, and a capillary tube (100 mm × 0.3 mm) was used to extract 1 μL of the solution for spotting to make a 5 × 3 olfactory visualization sensor array.

The data acquisition process of the sensor array was as follows: The accurately weighed rice sample of 20 g was placed in a beaker of 50 mL, the back of the sensor was attached to the plastic film, and the beaker was sealed with the plastic film to make the sensor react with the odor component produced by the rice sample for 30 min. In addition, a flatbed scanner (CanoScan Lide220, Canon, Tokyo, Japan) was used to scan the sensor images before and after the reaction.

In the study, firstly, the median filtering and the threshold segmentation were used to preprocess the original sensor image and the reacted sensor image. The average R, G, and B values of 15-pixel radii around each color-sensitive point were extracted and standardized to 0–255. Then, the three color components (i.e., ΔR, ΔG, and ΔB) could be obtained by making the difference between the reacted sensor image and the original sensor image. Finally, the ΔR, ΔG, and ΔB were normalized and then a difference image was generated by the recombined gray image. Three color components were obtained from each color-sensitive point, so the olfactory visualization sensor can extract 45 (3 × 15) color components for subsequent analysis.

#### 2.3.2. NIR Spectra Collection

The NIR spectra of all rice samples were collected using a portable NIR spectroscopy system built by the team. The system mainly included a spectrometer (NIRQuest512, Ocean Optics, Shanghai, China) and an integrating sphere (ISP-R, Ocean Optics, Shanghai, China). The specific collection process of the NIR spectra was as follows: The 20 g rice sample was poured into a quartz dish (to ensure that the bottom layer was covered), then the sample was covered with an integrating sphere, scanned in the wavelength range of 900–1700 nm, containing 512 data points, the integration time was set to 5 s, the average number of times was three, smooth degree was three. The original spectrum of the sample is the average of three measurements of the sample spectrum. During the spectrum collection process, the temperature in the laboratory was maintained at 25 °C. In this study, the original spectra were subjected to Savizkg-Golag (SG) smoothing and standard normal transformation (SNV) [33].

### 2.4. Multivariate Calibration Approaches

#### 2.4.1. Principal Components Analysis

Principal components analysis (PCA) is a technique for analyzing and simplifying data sets, and is a common method in multivariate analysis methods [34]. The basic principle is to find a spatial transformation according to the possible correlation between the initial feature quantities. By linearly combining the original variables, several new feature vectors are formed that are required to be orthogonal to each other and be able to maximize the retained original samples contained in the original information. At the same time, based on correlation requirements, several comprehensive variables are taken out to reflect the statistical methods of the original variables as much as possible [35].

In this study, the PCA was used to perform dimension reduction and feature mining on the 45 color components from the olfactory visualization sensor and the preprocessed NIR spectra.

#### 2.4.2. Backpropagation Neural Network

Backpropagation neural network (BPNN) is a supervised neural network learning algorithm, which uses the principle of error back propagation to change the traditional network structure and introduce new layers and logic [36]. The learning process consists of two parts: forward propagation and back propagation. In the forward propagation process, the input pattern is transmitted from the input layer to the output layer after being processed by hidden layer neurons. The neuron state of each layer only affects the neuron state of the next layer. If the desired output cannot be obtained in the output layer, then it will switch to back propagation. At this time, the error signal will propagate from the output layer to the input layer, and the connection weights and thresholds of each layer will be adjusted along the way. This process is repeated alternately until the global error of the network tends to a given minimum value. In the repeated training process, it uses the gradient descent method to make the weight change along the negative gradient direction of the error function and converge to the minimum point [37].

In this study, for BPNN, the hidden layer: three layers; the number of the hidden layer nodes: 5; the number of iterations: 100; the learning rate: 0.1; the minimum root mean square error: 0.00004. In addition, the results of 50 runs of the BPNN network were statistically analyzed to eliminate the influence of the randomness of parameter initialization on the model results. And the input feature of the BPNN is the principal components (PCs) extracted by the PCA.

## 3. Results

### 3.1. Trends of Fatty Acid Values

**Figure 2:**
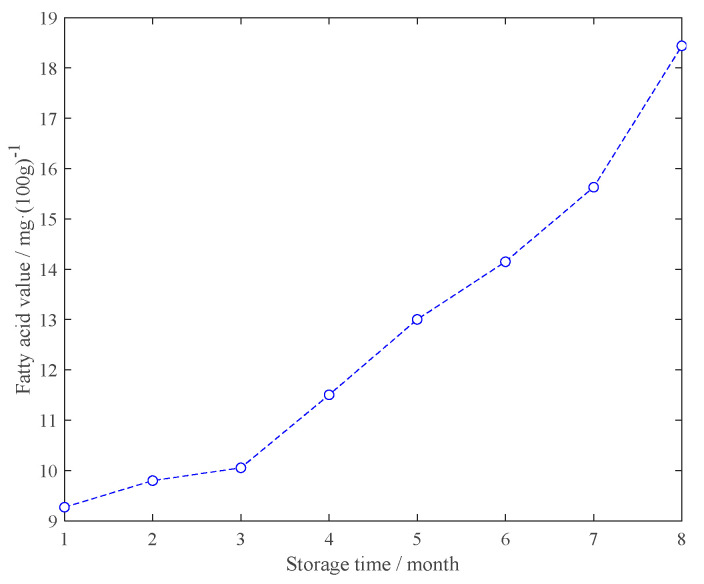
The variation trend of the fatty acid values of the rice samples in different storage periods.

Figure 2 shows the changes in the measured fatty acid values of the rice samples in different storage periods. It can be seen from Figure 2 that with the extension of the storage period, the fatty acid value in the rice shows an increasing trend, which directly reflects the changes in the internal components of the rice during the storage process.

### 3.2. Feature Description of NIR Spectra and Olfactory Visualization Sensor Data

From the NIR spectra of the rice samples in Figure 1, it can be seen that the useful absorptions for the full spectrum are at 1165, 1215 and 1395 nm, which are all bands associated with absorption CH groups in lipids, specifically, the CH second overtone region at 1165 and 1215 nm and CH combinations region at 1395 nm. Therefore, the absorption peaks of the NIR spectra have reflected the changes in the fatty acid values of the rice samples during the storage period.

**Figure 3:**
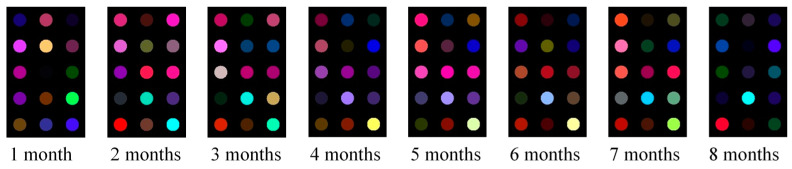
Differences in the olfactory visualization characteristics of rice samples over a monthly range of storage intervals.

Figure 3 shows the preprocessed images of the sensors of rice samples collected during different storage periods. It can be seen from Figure 3 that there are obvious differences between the image data of the rice samples, which directly shows that the composition and concentration of the odor produced by the rice samples in different storage periods are different. The prepared olfactory visualization sensor can reflect these changes. Therefore, the color features of the olfactory visualization sensor reflect the change of the fatty acid value of rice.

### 3.3. Sample Set Division

The sample division of the training set and the prediction set is for the training and construction of the BPNN model and external verification. In this study, 20 rice samples collected every month are randomly divided at a ratio of 3 to 1, and most of the samples after the division are put into the training set. Therefore, the training set ultimately contains 120 rice samples, while the prediction set contains 40 rice samples. Table 1 shows the results of the mathematical statistics of the fatty acid values in the two sample subsets.

### 3.4. Results of PCA

**Figure 4:**
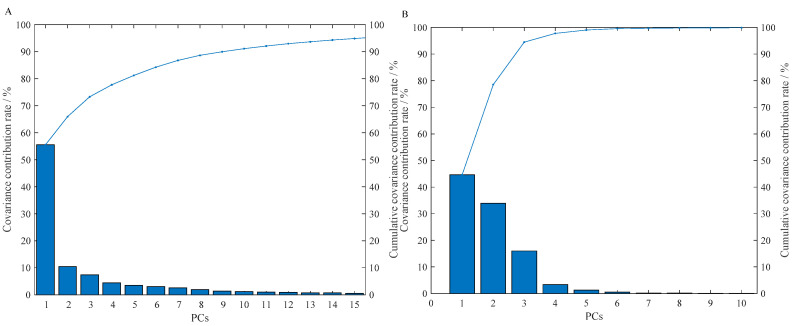
Statistical results of the covariance contribution rate and cumulative covariance contribution rate of the PCs with different technical data. (**A**) Olfactory visualization sensor; (**B**) NIR spectroscopy.

Figure 4A shows the statistical results of the covariance contribution rate and cumulative covariance contribution rate of each principal component (PC) after the color components of the olfactory visualization sensor are analyzed by the PCA. It can be seen from Figure 4A that the covariance contribution rate of the PC1 is the largest, 55.53%, indicating that it can explain 55.53% of the original variable information. After PC2, the covariance contribution rate is not very different, and gradually decreases, indicating that the degree of information reflecting the original variable is also gradually decreasing. Figure 4B shows the statistical results of the covariance contribution rate and cumulative covariance contribution rate of each PC of the preprocessed NIR spectra after the PCA. It can be seen from Figure 4B that the covariance contribution rate of the first three PCs is relatively large, which has explained most of the information of the full-spectrum data, and their cumulative covariance contribution rate reached 94.48%. From Figure 4, we can intuitively see the degree of interpretation and contribution of each PC to the original information.

### 3.5. Results of Quantitative Analysis

#### 3.5.1. Performance of BPNN Models Built on Olfactory Visualization Sensor Data

**Figure 5:**
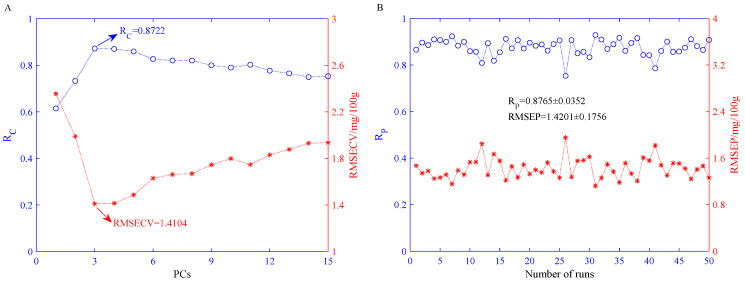
Statistical results of BPNN model based on olfactory visualization sensor data features after 50 independent runs. (**A**) Statistical results of BPNN models established under different PCs in the training set; (**B**) statistical results of the BPNN models established when PCs = 3 in the prediction set.

Figure 5A shows the result statistics of the BPNN models in the training set after running the BPNN 50 times. Figure 5A shows that when PCs = 3, the mean value of RMSECV reaches the minimum, which is 1.4104 mg/100 g, and the corresponding R_C_ mean value is 0.8722. Therefore, we believe that the number of the best PCs of the BPNN model is three. The statistical predictive results of the BPNN model constructed on three PCs in the prediction set are shown in Figure 5B. As can be seen from Figure 5B, the mean value of the root mean square error of prediction (RMSEP) is 1.4201 mg/100 g, and the mean value of the correlation coefficient of prediction (R_P_) is 0.8765. Their variances are 0.1756 mg/100 g and 0.0352, respectively.

#### 3.5.2. Performance of the BPNN Models Built on NIR Spectral Eigenvalues

**Figure 6:**
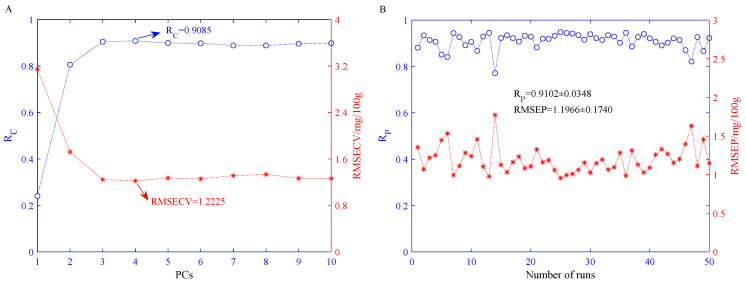
Statistical results of BPNN model based on portable NIR spectroscopy features after 50 independent runs. (**A**) Statistical results of BPNN models established under different PCs in the training set; (**B**) statistical results of the BPNN models established when PCs = 4 in the prediction set.

Figure 6A shows the statistical results of the R_C_ and the RMSECV in the training set after 50 independent BPNN runs with different PCs. As shown in Figure 6A, when PCs = 4, the mean value of RMSECV reaches a minimum of 1.2225 mg/100 g, and the mean value of the corresponding R_C_ is 0.9085. Therefore, we believe that the optimal number of PCs for the BPNN model based on the features of portable NIR spectra is four. When PCs = 4, the statistical predictive results of the BPNN model in the prediction set after 50 independent runs are shown in Figure 6B. Figure 6B illustrates that the average of the R_P_ is 0.9102 and the variance is 0.0348; the average of the RMSEP is 1.1966 mg/100 g and the variance is 0.1740 mg/100 g.

#### 3.5.3. Performance of the BPNN Models Built on Fusion Eigenvector

**Figure 7:**
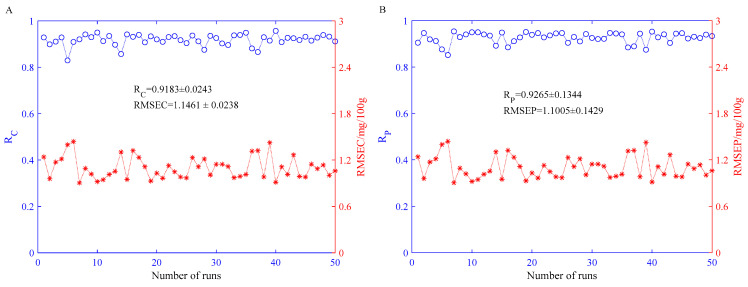
The statistical results of the BPNN models constructed on the fusion eigenvector. (**A**) Training set and (**B**) prediction set.

According to the previous analysis, the optimal number of PCs for the BPNN model based on olfactory visualization sensor data features is three, and the optimal number of PCs for portable NIR spectral features is four. In view of this, the study will fuse the best PCs obtained by optimization based on the BPNN models constructed on single technical data features at the feature layer and establish a fusion feature-based BPNN model to achieve quantitative detection of rice fatty acid content during storage. Figure 7 shows the statistical results of the BPNN model built on the fusion features after 50 independent runs. It can be seen from Figure 7A that the mean value of R_C_ is 0.9183 and the variance is 0.0243; the mean value of the root mean square error of calibration (RMSEC) is 1.1461 mg/100 g and the variance is 0.0238 mg/100 g. As can be seen from Figure 7B, the mean value of R_P_ is 0.9265 and the variance is 0.1344; the mean value of RMSEP is 1.1005 mg/100 g and the variance is 0.1429 mg/100 g.

### 3.6. Performance Comparison of Different BPNN Models

The detection performances of the best BPNN models based on different features are shown in Table 2. The correlation coefficients of BPNN models based on different data features are above 0.9. in both the training set and the prediction set This indicates that the quantitative detection of the fatty acid content of rice during storage can be achieved by the olfactory visualization sensor technology or the NIR spectroscopy technology. It is worth noting that, compared with the performance of the BPNN model based on the data features from the single technique, the BPNN models developed on the fusion eigenvector obtain better generalization performance and prediction accuracy. This shows that the BPNN model built on the fusion eigenvector can more fully reflect the internal and external quality characteristics of rice samples during storage. Therefore, the generalization and the performance prediction accuracy of the BPNN model based on the fusion feature are better.

## 4. Conclusions

The changes in physical and chemical indexes of rice during storage are caused by a combination of internal and external factors. The odor information of rice during storage can be effectively captured by the olfactory visualization sensors. Additionally, the NIR spectroscopy can quickly detect slight changes in the internal structure of the rice sample during the storage. This study has verified that the fusion of the olfactory visualization sensor characteristics and NIR spectral features can effectively improve the generalization performance and the prediction accuracy of the established chemometric model. The results obtained from this study can provide a high-precision technical method for the monitoring of grain quality during storage, in addition to providing significant practical protocols for ensuring grain storage quality.

## Figures and Tables

**Table 1:** The results of the mathematical statistics of the fatty acid values in the two sample subsets.

Subsets	Number of Samples	Minimum	Maximum	Mean	Standard Deviation
Training set	120	8.2997	21.0296	12.7229	3.1278
Prediction set	40	8.8247	20.5700	12.7565	3.0763

**Table 2:** The performance comparisons of different BPNN models.

Technique	PCs	Training Set		Prediction Set	
		R_C_	RMSE/mg/100 g	R_P_	RMSEP/mg/100 g
Olfactory visualization sensor	3	0.9224	1.1767	0.9103	1.2470
Portable NIR spectroscopy	4	0.9456	0.9490	0.9450	0.9767
Sensor fusion	7	0.9570	0.8564	0.9528	0.9123

## Data Availability

The data presented in this study are available on request from the corresponding author. The data are not publicly available due to the confidentiality requirements of the research project.
